# Fat deposition deficiency is critical for the high mortality of pre-weanling newborn piglets

**DOI:** 10.1186/s40104-018-0280-y

**Published:** 2018-08-27

**Authors:** Ting He, Long He, Enen Gao, Jinhua Hu, Jianjun Zang, Chunlin Wang, Jinshan Zhao, Xi Ma

**Affiliations:** 1 State Key Laboratory of Animal Nutrition, College of Animal Science and Technology, China Agricultural University, Beijing, 100193 China; 2 College of Animal Science and Technology, Qingdao Agricultural University, Qingdao, 266109 China; 3 Department of Internal Medicine, Department of Biochemistry, University of Texas Southwestern Medical Center, Dallas, TX 75390 USA

**Keywords:** Fat deposition, Mortality, Pre-weanling piglets, Thermogenesis, White adipose tissue

## Abstract

**Background:**

The high mortality of pre-weanling piglets is a dominant challenge which severely restricts the development of pig industry. A number of factors including nutrients imbalance and temperature variation during postnatal period of piglets have been reported to closely associated with the high mortality of postnatal piglets. This study aims to find out the relationship between fat deposition and survival of newborn piglets.

**Results:**

There were no differences in organ coefficient and bone density between the surviving and dead piglets (*P* > 0.05). The body weight and the fat deposition in the dead piglets were lower than the live individuals (*P* < 0.05). Consistently, the average sizes of white adipocytes in back and abdominal adipose tissues of dead piglets were smaller than the survivals (*P* < 0.05). The protein expression levels of adipocyte differentiation markers PPARγ and C/EBPα in the back and abdominal adipose tissues were lower in dead piglets compared to live piglets. The mRNA expressions of thermogenic markers *PGC1α* and *PRDM16* in adipose tissues were decreased in the dead piglets (*P* < 0.05). The microarray of back fat samples from the surviving and dead piglets were conducted; two down-regulated genes namely *AAMDC* and *CASTOR1* were identified from the dead piglets. According to quantitative real-time PCR (RT-PCR) analysis, the mRNA expression of *AAMDC* decreased, whereas *CASTOR1* expression elevated in the dead piglets compared to the surviving piglets (*P* < 0.05).

**Conclusions:**

The fat deposition and adipocyte differentiation in the dead piglets are insufficient compared to the surviving piglets, which may attenuate the thermogenic ability of white adipose tissue (WAT). Our data indicate that fat deposition in newborn piglets is vital to their survival.

## Background

In the modern pig industry, the mortality of piglet during suckling, weanling and nursery period accounts for a major portion until slaughter, of which the mortality reaches up to 16–20% from the phase of birth to weaning mainly due to stillbirth, crushing, hypothermia and starvation. Prolificacy of sow will cause the intra-uterine competition for nutrients and subsquently result in stillborn or live-born piglets with low birth weight [1].

The postnatal body weight of piglets largely determines the rate of survival. Study has found the piglets with a lower birth weight were more likely to die. The average size of piglets died postnatally was generally smaller than the live piglets and nearly 24.2% of the dead piglets have a shorter body length compared with the live ones [2].

Due to the physiological characters, newborn piglets are very vulnerable to the temperature fluctuation. The body temperature of piglets will suddenly drop by 2–4 °C after birth, while the process of temperature recovery usually consumes 24–48 h. Continuous hypothermia due to the low temperature condition and the nutrients competition will restrict the vitality and motility of the low-weight piglets [3]. Previous study found the average rectal temperature of newborn piglets that died in the first week after birth was 1 °C lower than that of the live ones [2]. The nutritional conditions especially the colostrum intake is not only important to maintain the immunity, but critical for the thermogenesis for the newborn piglets. It is found that the piglets with intrauterine growth restriction (IUGR) failed to suckle enough amount of colostrum and had lower temperatures at birth than piglets with normal body weight, which may lead to the high mortality of IUGR piglets [4].

Due to the absence of brown adipose tissue (BAT), the patterns of thermogenesis of newborn piglets mainly include the shivering thermogenesis and white adipose tissue (WAT) browning. Browning of WAT can stimulate the process of non-shivering thermogenesis as a potential strategy to facilitate the animal’s heat generation, and the beige adipocytes among WAT can be induced to produce heat while cold expose play key roles. It is reported that the acute cold stimulation could trigger browning of porcine WAT [5]. Thus, the WAT thermogenesis may be vital for piglets’ survival when in cold environment.

Adipocytes development begins at the fetal period. It was reported that the fetal fat deposition was 0.06 g/d during 0–69 d of gestation, and enhanced to 1.09 g/d after 69 d of gestation, indicating that the fat growth of fetus accelerated markedly after 69 d of gestation [6]. Neonatal piglets contain about 1% fat [7], while the fat content reaches up to 16.4% at postnatal 21 d, suggesting the rapid process of fat deposition in newborn piglets. However, the fat content of piglets fluctuates from 3% to 16% at 21 d weanling [7].

To date, whether the fat deposition deficiency is associated with higher pre-weaning piglet mortality remains unknown. This study aims to figure out the relationship between fat deposition and survival of pre-weanling piglets.

## Methods

### Animal model

A total of 20 Duroc × (Landrace × Yorkshire) crossbred newborn piglets derived from 10 litters were used in this experiment, including 10 dead piglets and 10 surviving littermates. All animal management and experimental procedures followed the animal care protocols approved by the China Agricultural University Animal Care and Use Ethics Committee. The ethics committee approval number is CAU20170625–2. During the experimental period, the lactating sows had free access to a corn-soybean meal-based diet that was formulated to meet NRC-recommended requirements (2012). Each sow freely nursed their piglets before weaning at 21 d of age.

### Experimental design and tissue collection

After sow farrowing, the neonatal piglets were nursed until weanling at the age of 21 d. During the lactating period, the fat content and bone density in the live and dead piglets were measured by dual energy X-ray absorptiometry (DXA) using a pencil beam scanner “GE Lunar DPX-IQ” with the whole- body mode “adult normal” (Lunar software version 4.7e, GE Healthcare, Pittsburgh, PA, USA) [8]. The live and dead piglets were weighed and then quickly dissected to collect the heart, liver, spleen, and kidney for weighing. The back fat and abdominal fat were collected, then quickly fixed in 4% paraformaldehyde or frozen in liquid nitrogen and stored at − 80 °C for subsequent analysis.

### Protein extraction and immunoblot analysis

The protein from back fat and abdominal fat tissue samples were extracted and detected by immunoblot described by Huang et al. [9]. Briefly, total protein was extracted according to the instruction of ProteoJET Total Protein Extraction Kit (Fermentas). The protein concentration was detected by Bicinchoninic Acid Protein Assay Kit (Applygen Technologies). Equal amounts of protein extracts (50 μg) were fractionated on 10% SDS-PAGE and transferred to PVDF membranes (Bio-Rad Laboratories). The membranes were blocked with a 5% skim milk solution at room temperature for 2 h and then incubated with corresponding primary antibodies: C/EBPα (1:1,000, Santa Cruz), PPARγ (1:1,000, Santa Cruz) and β-tubulin (1:1,000, Sigma) at 4 °C overnight. Thereafter, the membrane was washed 3 times with TBST and then incubated with the IRDye® 800CW-conjugated secondary antibody (1:10,000, LI-COR Bioscience). Fluorescence signals were visualized by the Odyssey LI-COR Biosciences Infrared Imaging System (Odyssey Software Inc., West Henrietta, NY).

### Histological measurement

The WATs were isolated and put into 4% paraformaldehyde buffer immediately and fixed for 24 h. Tissues were cut into about 1 cm in size, and then rinsed with running water for 1 h, and finally embedded in paraffin. Samples in paraffin were sectioned (4 μm), deparaffinized and re-hydrated. After that, tissue slides were stained with hematoxylin and eosin reagent. All the histological sections were observed and analyzed by polarizing microscope (BX-51, Olympus).

### Immunofluorescence

Firstly, paraffin embedded sections were deparaffinized and rehydrated, then washed with PBS and incubated with 0.1% Triton X-100 for 10 min, and then repaired the antigen in sodium citrate (pH 6.0) buffer by boiling 10 min in microwave oven. After cooling to the room temperature, sections were blocked in PBS containing 10% normal goat serum for 30 min at room temperature. Primary antibodies including PRDM16 (1:1.000, ab106410, Abcam) and PGC-1α (1:1,000, ab54481, Abcam) were then diluted in PBS and added to sections overnight at 4 °C. After washing in PBS, slides were then incubated with secondary antibodies that diluted in PBS containing 10% normal goat serum for 1 h at 37 °C. Slides were then mounted with Prolong Anti-Fade mounting medium containing DAPI (1:1,000, 28718–90-3, Sigma). All images were obtained using Leica SP5 scanning confocal microscope (Live cell Imaging Facility).

### Quantitative real-time PCR (RT-PCR)

Total RNA of WAT was isolated according to the instruction of TRIzol® reagent (Invitrogen Life Technologies) and genomic DNA was removed by treating RNA samples with the RNase-free-DNase-I (Fermentas, USA). Reverse transcription was performed with the ThermoSCRIPT RT-PCR System (Invitrogen, Carlsbard, CA) according to the manufacturer’s protocol. The RT-PCR was performed using the SYBR Green I dye kit (Roche Applied Science) on a TaqMan Sequence Detection System (Applied Biosystems). All reactions were run in triplicate. Relative gene expression was calculated with the comparative 2^−ΔΔCt^ method [10], using porcine *β-actin* as the reference gene. All primer sequences are listed: *AAMDC*: F: GAGAAAGGCGTGCAGATCCT R: CTTGGGTGACCAAGGCGTTA; *CASTOR1*: F: TCTCCCTCATCGAGGGCTAC R: TCCCACACTCATCAAAGCCC. *PGC1α*: F: GTCCTTCCTCCATGCCTGAC R: TGGTTTGCATGGTTCTGGGT; *PRDM16*: F: TACACGTGCAGGTACTGTGG R: GAGGTGTCTGTCCAGGTTGG; *β-actin*: F: TACACCGCTACCAGTTCGC R: GCTCGATGGGGTACTTGAGG.

### Microarray analysis

Total RNA of back fat was isolated and genomic DNA was removed according to the methods outlined above. The quantification of RNA was performed using spectrophotometer (ND-1000 NanoDrop, Wilmington, DE). The RNA then was prepared using the GeneChip (AFF-900623) one cycle target for the labeling and control reagents, and the labeled RNA was hybridized in an Affymetrix Hybridization Oven 640. After hybridization, the microarray slides were washed and dyed with the Affymetrix Fluidics Station 450. The probe arrays were scanned with an Affymetrix GeneChip Scanner 3000. The signal intensities of the spots on each image were quantified by the Affymetrix GeneChip Operating Software Version 1.4 (GCOS 1.4), and the data were adjusted and normalized by the dChip software. Microarray data was analyzed with BRB-ArrayTools software (Version3.7) developed by Dr. Richard Simon and BRB-Array Tools Development Team.

### Statistical analysis

Results are presented as means ± SEM. All data were analyzed using one-way ANOVA (the analysis of variance) procedure of SAS system (version 8.2, SAS Institute, Inc., Cary, NC, USA), followed by Fisher least significant difference test. *P* < 0.05 was considered statistically significant.

## Results

### Organ development parameters between the surviving and dead newborn piglets

The death of newborn piglets is probably related to organ developmental deficiency. Therefore, the heart, liver, spleen, and kidney of the surviving and the dead piglets were individually weighed and the organ coefficient was calculated. Results showed that the body weight of the dead piglets were lower than the surviving piglets (*P* < 0.05, Table 1). However, the organ coefficient and bone density were no differences between the two groups of piglets (*P* > 0.05, Table 1).

**Table 1:** Body weight, fat deposition, organ coefficient and bone density between the surviving and dead piglets

Items	Surviving piglets	Dead piglets	*P*-value
Body weight, kg	6.93 ± 1.62^a^	5.13 ± 1.42^b^	< 0.05
Fat deposition, %	19.6 ± 1.3^a^	14.2 ± 2.1^b^	< 0.05
Organ coefficient, %	22.4 ± 3.9	23.6 ± 4.5	> 0.05
Bone density, g/cm^2^	17.5 ± 2.0	18.4 ± 3.0	> 0.05

^a, b^Different letters in the same row means significant differences, value are means ± SEM. *n* = 10

### Deficiency of fat deposition and white adipocyte differentiation in the dead piglets

**Fig. 1:**
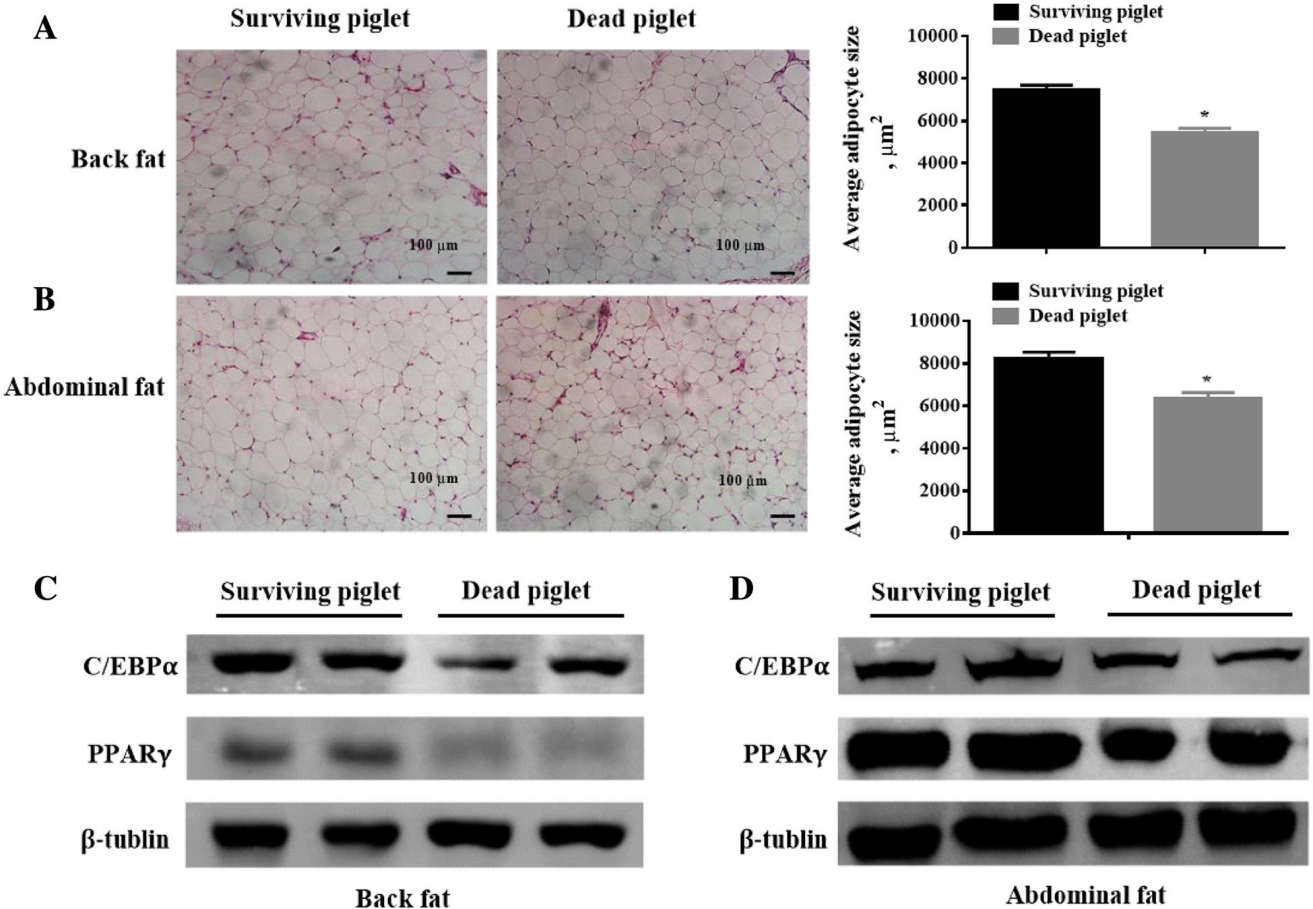
The fat deposition and white adipocyte differentiation between the surviving and dead piglets. **a**-**b**, The average size of white adipocytes in both back and abdominal fat tissues of dead piglets were smaller than the surviving pigs. * *P* < 0.05; **c**-**d**, The expressions of PPARγ and C/EBPα in both back and abdominal fat tissues were less in the dead piglets than the survivals. * *P* < 0.05, *n* = 10

Owing to the lighter body weight and the unchanged organ coefficient and bone density in the dead piglets compared with the living siblings, we speculated that the WAT mass might be less in the dead pigs. Consequently, we measured the back fat and abdominal fat contents of piglets and verified that the fat deposition in dead piglets were less than the live piglets (*P* < 0.05, Table 1). The average size of white adipocytes in back and the abdominal fat tissues were measured. The white adipocyte size of dead piglets was smaller than the surviving piglets (*P* < 0.05, Fig. 1a-b). The smaller size of adipocytes might be related with the insufficient differentiation of pre-adipocytes. Therefore, we measured the protein expressions of mature white adipocyte markers PPARγ and C/EBPα. The protein expression levels of PPARγ and C/EBPα in both back and abdominal fat tissues were lower in the dead piglets than the survivals (*P* < 0.05, Fig. 1c-d).

### Deficiency of WAT thermogenesis in dead piglets

**Fig. 2:**
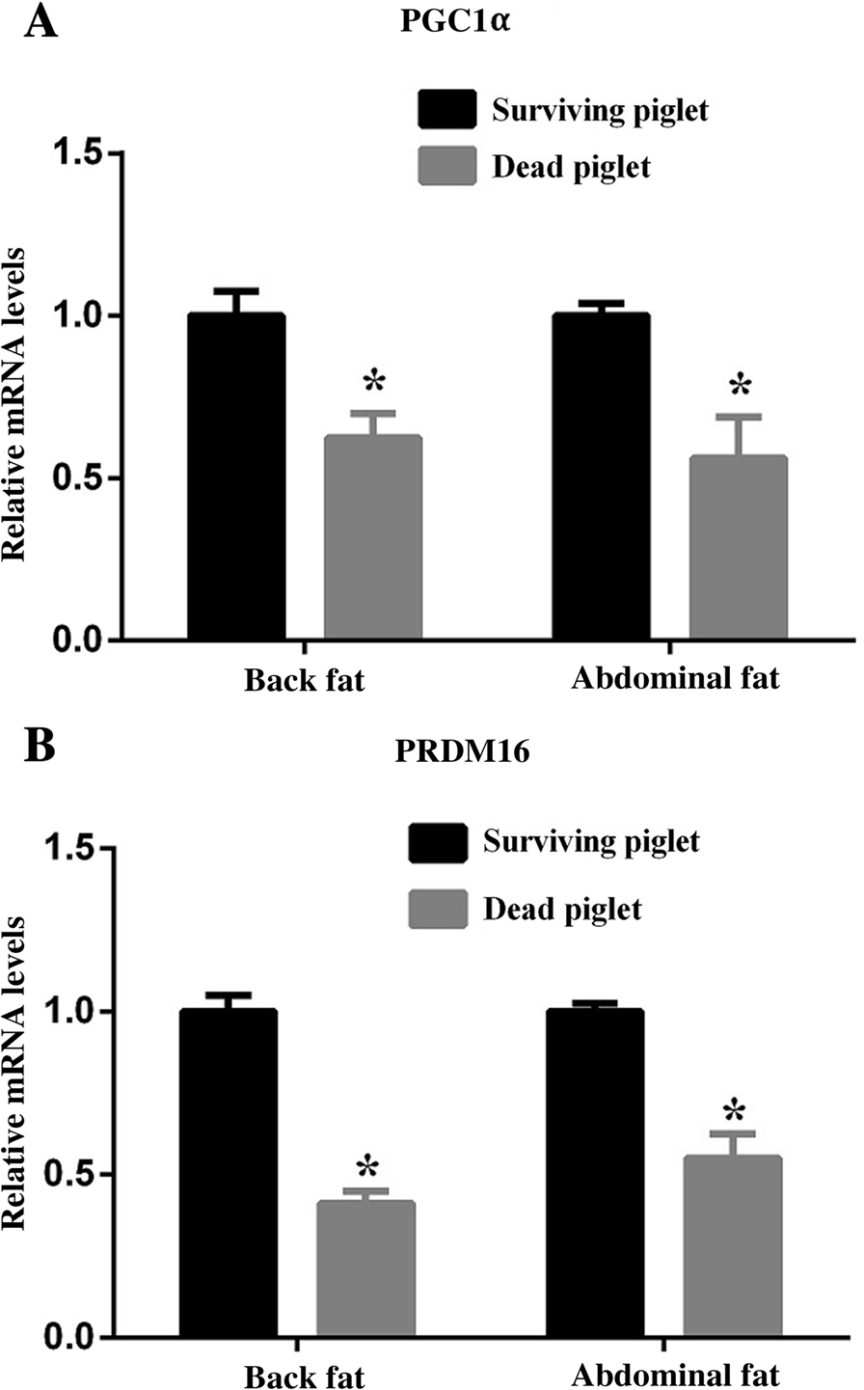
The mRNA expression of *PGC1α* and *PRDM16* in back and abdominal fat tissues between the surviving and dead piglets. **a**-**b**, The relative mRNA levels of *PGC1α* and *PRDM16* were elevated in both fat pads in surviving piglets compared to the dead piglets. *P < 0.05, *n* = 10

**Fig. 3:**
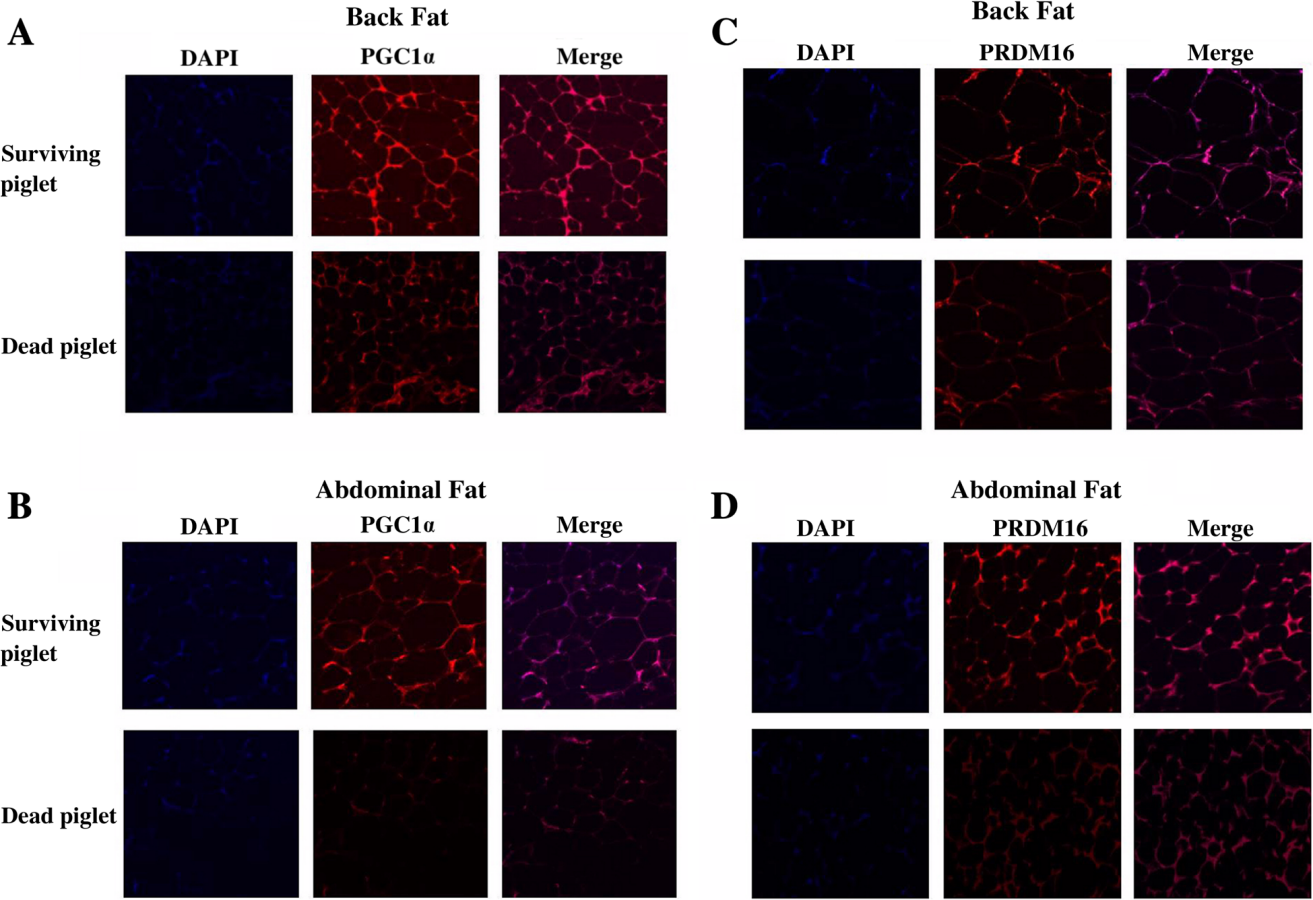
The immunostaining of *PGC1α* or *PRDM16* in back and abdominal fat tissues between the surviving and dead piglets. **a**-**d**, The *PGC1α* and *PRDM16* expressions in both back and abdominal fat tissues were higher in living piglets than dead piglets

The newborn piglets need to conquer the challenge of low environmental temperature via mobilizing the thermogenesis process. The lack of thermogenesis might be fatal for the neonate. Accordingly, we measured the thermogenic genes in the back and abdominal fat tissues. The relative mRNA expression of *PGC1α* and *PRDM16* in both back fat and abdominal fat were decreased in the dead piglets compared to the surviving piglets (*P* < 0.05, Fig. 2). The result of immunostaining of WAT also showed the expressions of PGC1*α* and PRDM16 were lower in the dead piglets than survivals (*P* < 0.05, Fig. 3).

### Microarray-base screen identified genes might be involved in the fat deposition between dead and surviving piglets

**Fig. 4:**
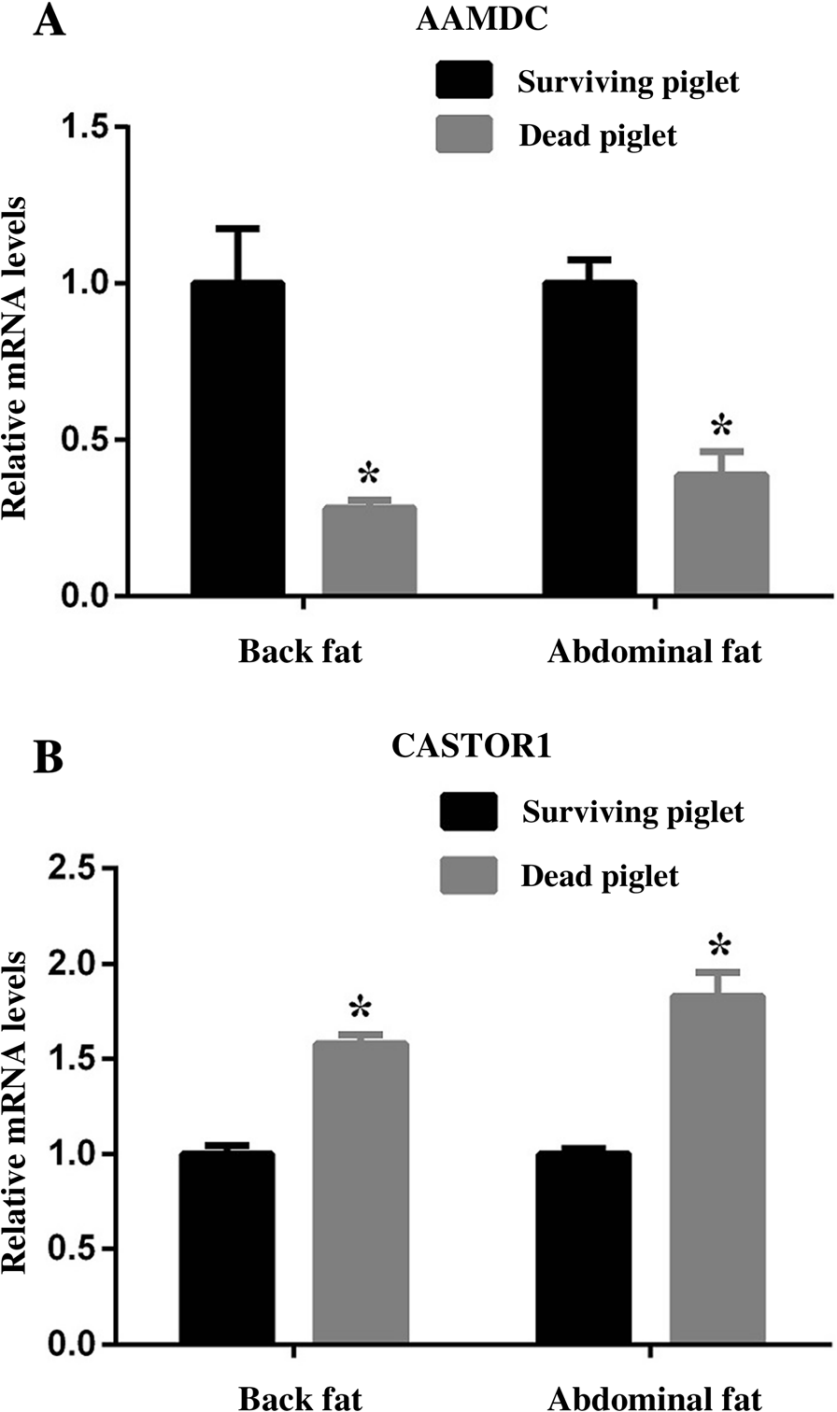
Candidate genes involving in the adipose tissue development between the surviving and dead piglet were screened out. **a**, The relative mRNA level of *AAMDC* was elevated in both fat pads in surviving piglets compared to the dead piglets. **b**, The relative mRNA level of *CASTOR1* was decreased in both fat pads in surviving piglets compared to the dead piglets. * *P* < 0.05, *n* = 10

To figure out the underlying mechanism of fat deposition in dead and surviving piglets, microarray was conducted to screen the potential genes participated in this process. Two genes namely *AAMDC* and *CASTOR1* were identified. The RT-PCR verified that the mRNA expression of *AAMDC* was decreased in the WAT of dead piglets than survivals (*P* < 0.05, Fig. 4). However, the mRNA expression of *CASTOR1* was elevated in the dead piglets (*P* < 0.05, Fig. 4).

## Discussion

The mortality of newborn piglets varies between 12% and 25% due to complicated reasons, such as nutrients supplementation for lactating sow, weight of litter, temperature and management during the pre-weanling stage [11–13]. High mortality of pre-weanling piglets result in a large economy loss and resources waste in pig industry. Piglets with low birth weight less than 1.13 kg are at risk of lower survival rate and longer time to market [14]. Previous study has proposed the relationship between birth weight and survival rate for piglets, when increased birth weight class by 100, 150, and 200 g; the survival rate of piglets would increase to 85.6%, 86.6%, and 87.6%, respectively; and time to reach 100 kg would decrease from 178.7 d to 176.1 d, 174.5 d, and 172.9 d, respectively [14]. Another study has also demonstrated that the lower littermate weight was associated with higher mortality [15]. Consistently, we have also found that the body weight of the dead piglets was lower than the surviving piglets, indicating that the low body weight of newborn piglets is closely associated with the high risk of dying before weanling. However, death still occurred in the piglets with normal body weight. Therefore, there exist other important factors affecting the mortality of preweaning piglet except for the body weight.

We therewith measured the organ coefficient and the bone density of piglets to investigate whether the death of piglets are related to abnormal organ development. Although the organ coefficient and the bone density showed no differences between the dead and surviving piglets in this study, the fat content of dead piglets was significantly lower than the survivals. Thus, our attention has been shifted to the role of adipose tissue on pre-weanling piglet survival.

The adipose tissue distributes throughout the body and generally functions as a specialized energy deposition pool to form lipid droplet [16]. To evaluate the impacts of adipose tissue on the mortality of pre-weanling piglets, we measured the size of adipocytes and the protein expressions of adipocyte differentiation markers in back and abdominal fat tissues of piglets. The sizes of white adipocytes in dead piglets were smaller than the surviving pigs and the protein expressions of PPARγ and C/EBPα were lower in the dead piglets than the survivals. These results demonstrated that the general low body weight of dead piglets may attribute to the deficiency of fat deposition and adipocyte differentiation. Usually, the development of adipose tissue in early postnatal piglets is considered to link with maternal nutrition condition. It is reported that the expression of PPARγ in subcutaneous adipose tissue was upregulated in piglets orally gavaged with butyrate [17], a four-carbon short-chain fatty acid which has been widely reported with its beneficial effects on intestinal homeostasis and energy metabolism [18]. Moreover, the piglets born to sows that were fed with butyrate during the last trimester of pregnancy have a significantly increased body weight than individuals without butyrate feed [17]. Another study demonstrated that both of the low (below 50%) and high (above 250%) intake of dietary protein for gilts compared with the normal supplemetation during gestation would reduce body fat content in newborn piglets [19]. Moreover, suckling piglets explosed to a high protein formula represents a transitory reduction in adipose tissue development at weaning compared with pigs with adequate protein content [20]. Accordingly, the nutrition supply for newborn piglets as well as the dietary formula of lactating sows are greatly involved in the modulation of the adipose tissue development in piglets and which might be vital to the piglet survival.

The body temperature of piglets decreased shortly after birth, which is known as postnatal hypothermia. The extent and duration of this postnatal hypothermia is considered to negatively correlate to the survival rate of newborn piglets [11]. Due to the lack of BAT in piglets, thermogenesis in WAT becomes essential for maintenance of body temperature. The thermogenic pattern of WAT changes according to the environment temperature. The acute cold exposure (4 °C, 10 h) could activate WAT thermogenesis by elevating thermogenic genes expression. Conversely, chronic cold exposure (8 °C, 15 d) inhibited the WAT thermogenesis [5]. Accordingly, continuously cold stimulation may destroy the balance of thermogenic regulation of WAT and eventually cause the death of piglets. We speculated that the thermogenesis of WAT in dead piglets were weaker than the surviving piglets due to the developmental deficiency of WAT. As a result, the relative mRNA expressions of thermogenic genes *PGC1α* and *PRDM16* in fat tissues of dead piglets were significantly lower than the survivals. Consistently, the analysis of immunostaining of PGC1*α* and PRDM16 demonstrated the less thermogenesis of WAT in dead piglets.

Microarray was previously conducted to screen the potential genes which may be involved in WAT development, and two candidate genes: *AAMDC* and *CASTOR1* were screened. The relative mRNA expression of *AAMDC* in the back adipose tissue was lower in the dead piglets compared to the surviving ones. Conversely, the relative mRNA expression of *CASTOR1* was higher in the dead piglets.

Aamdc highly expresses in white adipose tissue and has been identified as an activator of adipogenesis through upregulation of CREB transcription activity [21, 22]. Overexpression of the Aamdc protein promotes adipogenic differentiation of 3T3-L1 preadipocytes. Conversely, depletion of the Aamdc blockes the process of insulin-induced 3T3-L1 differentiation [21]. Interestingly, CASTOR1 is one of mTORC1 negative regulator which has been identified as cellular arginine sensor [23]. Study has revealed that the mTORC1 pathway is essential for adipocyte differentiation. Activation of mTORC1 would phosphorylate S6K1 to upregulate the expressions of PPARγ and C/EBPα and promote adipogenesis [24]. The lower expression level of *AAMDC* in the dead piglets’ WAT indicated the deficiency of adipogenic differentiation, which was consistent with the previous results [21]. The elevation of *CASTOR1* expression in WAT may inhibit the mTORC1 pathway, which leads to inhibition of adipogenesis in the dead piglets. However, the underlying mechanisms of AAMDC and CASTOR1 mediated adipocytes’ differentiation are needed to be further explored.

## Conclusions

In conclusion, the deficiency of fat deposition as well as decreased thermogenic ability of WAT are positively related to high mortality of pre-weanling piglets, in which the *AAMDC* and *CASTOR1* genes may play vital roles in regulating the process of fat deposition of newborn piglets.

## Abbreviations

BAT: Brown adipose tissue
DXA: Dual energy X-ray absorptiometry
IUGR: Intrauterine growth restriction
RT-PCR: Quantitative real-time PCR
WAT: White adipose tissue
